# Quality Principles of App Description Texts and Their Significance in Deciding to Use Health Apps as Assessed by Medical Students: Survey Study

**DOI:** 10.2196/13375

**Published:** 2019-02-27

**Authors:** Urs-Vito Albrecht, Christin Malinka, Sarah Long, Tobias Raupach, Gerd Hasenfuß, Ute von Jan

**Affiliations:** 1 Peter L Reichertz Institute for Medical Informatics Hannover Medical School Hannover Germany; 2 Department of Cardiology and Pneumology University Medical Center Göttingen Göttingen Germany; 3 Division of Medical Education Research and Curriculum Development University Medical Center Göttingen Göttingen Germany

**Keywords:** mobile health, evaluation studies, mobile applications, quality criteria, usage decision

## Abstract

**Background:**

Currently, there are no binding requirements for manufacturers prescribing which information must be included in the app descriptions of health apps.

**Objective:**

The aim of this study was to investigate how medical students perceive a selection of quality principles, intended for usage decisions in the app context, and establish whether the information presented in a sample of app descriptions is perceived as sufficient for facilitating an informed usage decision.

**Methods:**

A total of 123 students (mean age 24.2 years, SD 3.4) participating in a 6-week teaching module covering cardiology and pulmonology at the University of Göttingen (original enrollment 152 students, response rate 80.9%) were included. Students were asked to read 3 store description texts of cardiological or pneumological apps and initially assess whether the descriptions sufficed for a usage decision. Subsequently, they were queried on their perception of the relevance of 9 predefined quality principles, formulated for usage decisions. An appraisal of whether the app description texts contained sufficient information to satisfy these quality principles followed. By means of 20 guiding questions, participants were then asked to identify relevant information (or a lack thereof) within the descriptions. A reassessment of whether the description texts sufficed for making a usage decision ensued. A total of 343 complete datasets were obtained.

**Results:**

A majority of the quality principles were described as “very important” and “important” for making a usage decision. When accessed via the predefined principles, students felt unable to identify sufficient information within the app descriptions in 68.81% (2124/3087) of cases. Notably, information regarding undesired effects (91.8%, 315/343), ethical soundness (90.1%, 309/343), measures taken to avert risks (89.2%, 306/343), conflicts of interest (88.3%, 303/343), and the location of data storage (87.8%, 301/343) was lacking. Following participants’ engagement with the quality principles, statistically significant changes in their assessment of whether the app descriptions sufficed for a usage decision can be seen—McNemar-Bowker test (3)=45.803919, *P*<.001, Cohen g=.295. In 34.1% (117/343) cases, the assessment was revised. About 3 quarters of changed assessments were seen more critically (76.9%, 90/117). Although, initially, 70% (240/343) had been considered “sufficient,” this rate was reduced to 54.2% (186/343) in the second assessment.

**Conclusions:**

In a considerable number of app descriptions, participants were unable to locate the information necessary for making an informed usage decision. Participants’ sensitization to the quality principles led to changes in their assessment of app descriptions as a tool for usage decisions. Better transparency in app descriptions released by manufacturers and the exposure of users to quality principles could collectively form the basis for well-founded usage decisions.

## Introduction

### Background

The market for health apps, that is, health-related apps running on mobile devices such as smartphones and tablet computers, is highly liberal and poorly regulated. This not only facilitates the creation of software, resulting in a large supply but also immensely influences user access and app usage. In this climate, we observe a flood of health apps, market dynamics typical for apps, and an associated lack of commitment to quality control [1]. From a government perspective, comprehensive (cross-border) monitoring of the market proves almost impossible [2,3]. Other entities (existing and emerging private and scientific testing or certification initiatives) [4-13] have yet to prove their efficiency and suitability for mapping the market [14]. The ultimate responsibility for deciding to utilize mobile apps rests with the users and cannot be transferred. In the context of health, this has even further-reaching implications than in other areas. Here, apps are used by laypersons as well as medical professionals in a highly sensitive environment. Apps, and the technology used to run them, are designed to be fully integrated into the user's everyday life. This aspect offers the greatest possible user comfort in both private and professional settings. Despite this unique advantage, it is important to recognize and respect certain legal boundaries, particularly addressing laws concerning medical practitioners [15]. These boundaries exist to protect both doctors and their patients and apply to using or recommending apps. In Germany, for example, laws cover confidentiality, advertising regulation, and the patient's freedom of choice concerning methods in diagnostics and therapy, given that these are appropriate and correspond to the current state of technological and scientific progress. These factors must be guaranteed by the medical staff as guarantors for their patients [16]. If applicable, rules are not followed, leading to damage infliction, and this is facilitated by a recommended or utilized app, medical staff involved can be held liable [16,17]. Consequently, doctors and other health professionals must (ethically and legally) inform themselves, undertaking a case-by-case risk-benefit assessment before recommending, or themselves deciding to use health-related apps. At the outset, similar to users with other backgrounds, medical professionals will likely—at least initially—rely on App Store description texts when selecting an app. Other information or test results and quality seals and the like are not often readily and reliably available [14] without (greater and time-consuming) research effort, or their reliability may be questionable because of various reasons. For this to be effective, it is imperative that manufacturers provide transparent information about their apps. Such transparency can serve as a reasonable basis for usage decisions. Thus, high-quality and trustworthy software has a better chance of asserting itself, and the self-regulatory capacity of the market can be supported [18].

Ideally, decisions for or against the use of an app are made by the interested parties who know their individual requirements best [19] and base their decisions on comprehensive information from multiple sources. A wide variety of tools and guidelines have been and are being developed on the basis of this principle [6,20-32], all of which share the common goal of supporting users in the decision process. In particular, there is a focus on requirements in the precarious context of health and medicine [7,33-40], taking into account both possible benefits and potential risks [41]. Many of these, for example, are published in the form of checklists that users may apply to the apps they are interested in [8,39], usually after installing them. However, it is currently almost impossible to estimate the extent to which the information available in the stores (in the form of app descriptions) can be used to adequately assess the suitability of an app before use. Existing studies, which also investigate the role of app descriptions, tend to focus on facets other than usage decisions, such as aspects related to marketing (and thus turnover-relevant aspects), rather than attempt to examine the quality of the content in serving its purpose [42]. With regard to app security, store description texts are used by researchers to compare the actual behavior of apps, for example, in the context of data transfers or potentially harmful functions (integration of advertising networks, etc), with the information contained in the descriptions [43,44].

### Objectives

Supplementing gaps in existing research, in this study, the following questions were investigated: (1) which quality principles students consider fundamentally relevant for making a usage decision? (2) Whether or not the information in the submitted app descriptions is perceived as sufficient for a usage decision, (3) whether or not quality aspects can be identified within the description texts using key questions, and (4) whether or not exposure to the quality principles provokes a change in the students’ assessment.

## Methods

### Setting

The study took place in the autumn of 2018 as part of a 6-week teaching module in the clinical phase of the undergraduate medical education program at the University of Göttingen, Germany. Within this module, a 6-hour practical training module was introduced, in which fourth-year students had the opportunity to explore health-related apps. The students had the opportunity to volunteer their data for this study. Nonparticipation would not have had any effect on the successful completion of the course. The students were informed in advance and were asked for their consent. The study was approved by the local Ethics Committee (application number 18/9/18), and all participants provided written consent.

For the purpose of this study, the Web-based survey system EvaSys (version 7.1, Electric Paper Evaluationssysteme GmbH, Lüneburg, Germany) was used; the surveys were pseudonymized. In the first step, students were asked to provide demographic information. Each participant was then randomly assigned 3 app descriptions from a pool of health apps from the fields of cardiology and pulmonology, which were compiled by applying the keyword-based Semiautomated Retrospective App Store Analysis (SARASA) filtering processes to a readout of apps listed in the “Medical” category of Apple’s App Store in August 2018 [45]. A wide range of apps for both patients and medical professionals was selected for the study. Examples of these include reference and learning apps as well as health diaries, treatment plans, and calculators. During the seminar, each student independently examined the app descriptions assigned to him or her in a multistep process.

**Figure 1:**
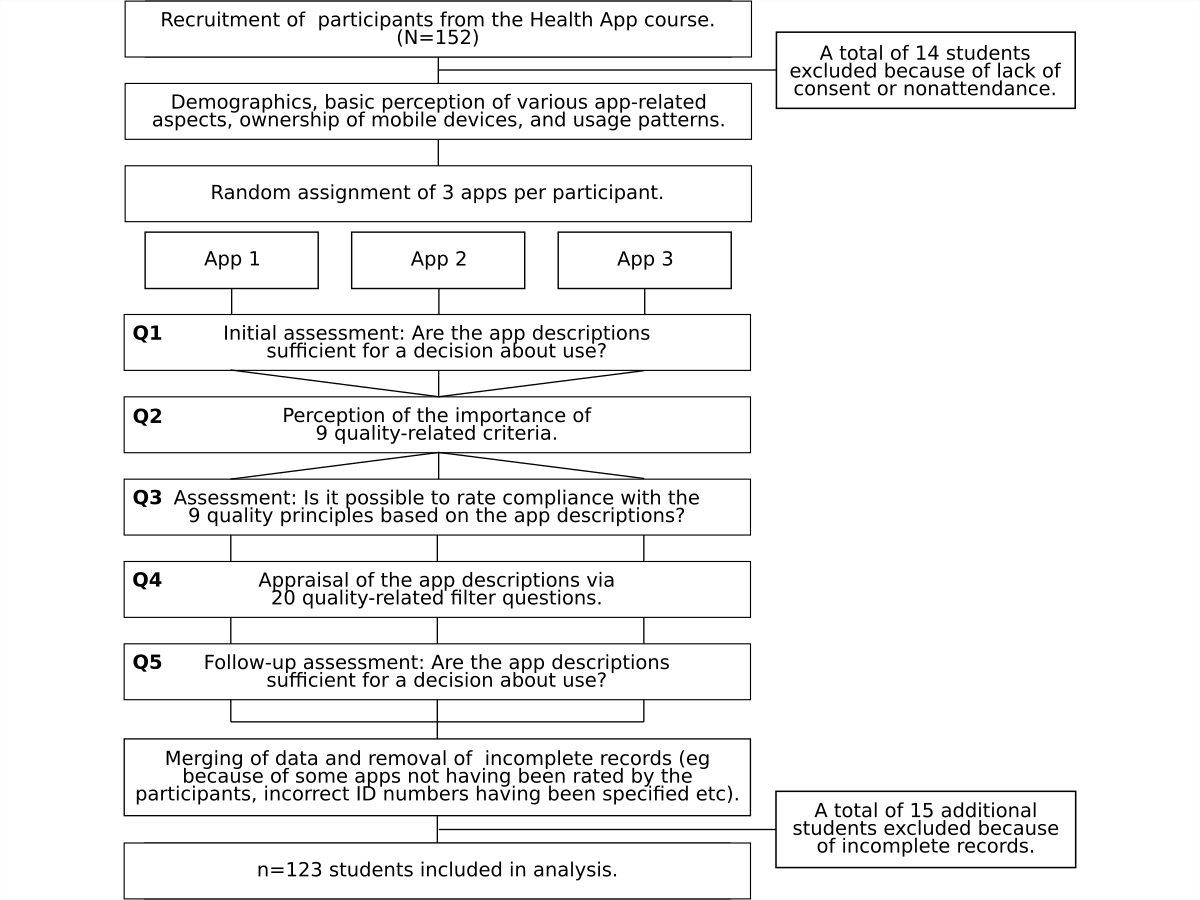
Study design and procedure.

After having provided basic demographic information, the students were asked for their initial assessment (not yet influenced by discussions, explanations, or having explored the quality criteria) of whether the app descriptions provided sufficient information for a decision on use (“The app description is sufficient for me to make a decision on use,” “The app description is not sufficient for me to make a decision on use,” or “I don't know”; see question block Q1, Figure 1). Immediately after this evaluation, the students were asked to express their—still uninfluenced—opinion on the importance of 9 quality principles for their usage decision—see definitions in subsection “Quality Principles and Operationalization” below, predominantly based on International Organization for Standardization/International Electrotechnical Commission (ISO/IEC) 25010 [46], question Block Q2, Figure 1, stratified by “very important,” “important,” “part/part,” “less important,” “unimportant,” and “do not know.”

Again, without explanations, the students were then requested to indicate whether the app descriptions provided allowed an assessment of the individual quality principles (see Q3, Figure 1). Subsequently, they were presented with 20 questions to be answered with “yes,” “no,” or “do not know” on the actual content of the app descriptions (eg, information on the purpose of the app, fields of application, target groups of the apps, or the respective providers; see section Quality Principles and Operationalization). These were based on the items presented in other studies [35,47] (see Q4, Figure 1) and covered aspects related to the 9 quality principles in the hope that working with these questions would increase participants’ awareness of aspects related to these quality principles. Unfortunately, the question “Is there information about the aptitude (qualification) of the authors/developers of the app?” was not incorporated in the electronic survey, but for the sake of completeness, it is still listed in the section Quality Principles and Operationalization. Finally, the students were again prompted to assess whether, in their opinion, the app descriptions contained sufficient information for a decision on use (see Q5, Figure 1).

**Table 1:** Demographics for the participants.

Characteristics		Male (n=42)	Female (n=80)	Unspecified (n=1)	Total (n=123)
Age, mean (SD)		24.8 (3.2)	23.9 (3.4)	23 (—^a^)	24.2 (3.4)
Years of study, mean (SD)		4.1 (0.4)	4.1 (0.4)	4 (—)	4.1 (0.4)
**Mobile operating system (corresponding number of participants, n)**					
	iOS (tablet, smartphone, or iPod)	24	47	1	72
	Android (tablet or smartphone)	21	36	0	57
	Other (tablet or smartphone)	2	2	0	4
	Several different OS (accumulated)	4	5	0	9
**Use of apps in general (corresponding number of participants, n)**					
	No	4	2	1	7
	Yes	38	78	0	117
Ratings submitted (total, N=343)		115	225	3	343
Ratings provided (per participant, mean [SD])		2.7 (0.6)	2.8 (0.4)	3 (—)	2.8 (0.5)
Apps assigned (n)		90	132	3	143

^a^Not applicable.

### Study Population

Of a gross total of 152 medical students who had registered for the class, those who did not attend the course despite registration or did not give their consent (n=14) were not included in the study; thus, 138 participants in their fourth academic year remained (Table 1). The evaluation only included complete datasets. Participants’ responses to the various parts of the survey (Figure 1) could be linked via their individual identification number and the name of the respective app. By answering all questionnaires for at least 1 of the 3 apps (selected from a set of 143 individual apps) assigned to them, participants qualified their data for inclusion in the analysis. Thus, a total of 343 app-related assessments (82.9% of 414 expected, dropout: 15 students) from 123 students (89.1%, 123/138) remained (42 males, 80 females, gender not indicated in 1 case, overall mean age 24.2 years, SD 3.4). Of these 123 students, not all completed all question blocks for all 3 of their assigned apps (or it was impossible to match these to a specific app or student, eg, because of errors typing identification numbers), resulting in an average of only 2.8 app evaluations per participant available for evaluation (SD 0.5).

Only 7 participants stated that they do not use any apps. Mainly iOS-based smartphones and tablets were in use (72/123 respectively 58.5% total, males: 24/42 respectively. 57.1%, females: 47/80 respectively. 58.8%), followed by Android-based mobile devices (57/123 mentions respectively. 46.3% total, males: 21/42 respectively. 50%, females: 36/80 respectively. 45%). With the exception of gender, the study population included in the evaluation is homogeneous. Approximately twice as many females were included, as opposed to males. This reflects the larger proportion of female students documented undertaking a medical degree at the University of Göttingen and at German universities in general [48]. A correlation between the evaluations of the app descriptions and participants’ gender could not be shown—Pearson Chi-square χ^2^_4_=8.4, *P*=.77, n=123).

### Quality Principles and Operationalization

The study was focused on 9 quality principles (see Table 2), predominantly modeled on ISO/IEC 25010 [46] for health software, that are currently being discussed in the context of coordinating interdisciplinary quality criteria in Germany, (eg, as compiled by Albrecht [49,50]). Although it could be argued that other criteria could also have been included in this study, we explicitly chose not to do so, as these proved to be too specific to be appropriate for the assessments we had planned. For example, although ISO/IEC 25023 [51] provides a “basic set of quality measures” for various quality aspects and “an explanation of how to apply software product and system quality measures,” we chose not to include it, as the purpose of the part of the study presented here was not to measure app quality but rather to analyze participants’ subjective perceptions of quality, via app descriptions.

Additional sources were used to support the compilation of the 9 principles; however, no single source was fully adopted, for reasons also noted by Nouri et al [40] in their study on quality principles in the app context.

There is hardly any agreement among different working groups or authors as to which quality categories and characteristics can be usefully applied to an assessment or which characteristics can be assigned to which quality categories and how it should be determined whether an app offers the desired characteristics. This can be illustrated exemplarily by the aspect of usability [3], but it can also be established in principle for all other areas relevant in the quality context. Differences exist, among other things, with regard to the assignment of different characteristics to the usability principle, but this may also be because of different objectives or target groups such as consumers or the restriction to selected application areas of the respective approaches. Objective as well as more subjective characteristics are often included. Although Zapata et al [52], for example, included rather subjectively assessable aspects such as attractiveness, learnability, usability, and comprehensibility in their empirical analysis on usability, other authors approach the concept of usability from a technical and more objective point of view. Brown et al [53] did this by subdividing the usability of the “Health IT Usability Evaluation Model” presented in another study [54] into more detailed parts such as avoidance, completeness, memory, need for information, flexibility/adaptability, learnability, speed of performance, and competence. Nevertheless, in some cases, the various characteristics can be difficult to assess without in-depth technical and/or content-related knowledge or in some cases, time-consuming analyses. It is for this reason that, in our operationalization of the 9 quality principles, we tried to keep the questions the students were confronted with simple to comprehend and easy to answer, still addressing the quality principles without going into great technical detail. The operationalization itself (Table 3) was done by comparing the quality principles with existing question lists for self-assessments of health apps from the preliminary study conducted both internally and also in accordance with several other German initiatives [19,35,37].

**Table 2:** The 9 quality principles (predominantly based on ISO 25010, with supporting sources also listed).

Quality principle	Description	(Sub) Section of ISO/IEC^a^ 25010 [46]	Supporting sources
Practicality	High-quality software must be flexible enough to be used for the intended purpose and, if possible, beyond it, to cover the widest possible range of use and application contexts.	4.1.3 satisfaction; 4.1.5 context coverage; 4.2.1 functional suitability; 4.4.11 stated purpose.	[35,37,38,55-64]
Risk adequacy	It must be possible to use software in a risk-appropriate manner without exposing the user or his or her environment to unreasonable health, social, or economic risks.	4.1.4 freedom from risk (economic, health and safety, and environmental risk mitigation).	[55,56,65,66]
Ethical soundness	Development, provision, operation, and use must be ethically innocuous to prevent discrimination and stigmatization and to provide fair access.	4.2.4.6 accessibility	[37,38,56,62,67,68]
Legal conformity	The legal conformity (eg, with regard to medical device law, professional codes of conduct, data protection laws, laws on the advertising of therapeutic products) for development, provision, operation, and use must be guaranteed for the protection of all parties involved (eg, providers, store operators, and users).	—^b^	[9,19,35,37,38,55,56,61,69-73]
Content validity	The content presented and used must be valid and trustworthy.	—^c^	[8,9,19,38,40,56,59-62,73-76]
Technical adequacy	Development, operation, and use need to be appropriately adapted to the capabilities of the technology and the current state-of-the-art to ensure sustainability in terms of maintainability, portability, interoperability, and compatibility.	4.2.3 compatibility; 4.2.5 reliability; 4.2.7 maintainability, and 4.2.8 portability	[40,66,73,77]
Usability	The software must have a high degree of usability appropriate for its target groups, that is, it must be user-friendly and easy to use, taking into account the relevant circumstances and conditions. This can facilitate fair and sustainable use that is also convenient and contributes to user satisfaction.	4.1.3.4 comfort; 4.2.4 usability; 4.2.8.1 adaptability.	[40,73,78]
Resource efficiency	Elements for resource-efficient operation and use should be taken into account during development.	4.1.2 efficiency and 4.2.2 performance efficiency (including time behavior, resource utilization, and capacity)	[65,73]
Transparency	Full transparency regarding the aforementioned criteria serves as a basis for software evaluations as well as for individual and collective usage decisions.	—^c^	[9,19,35,66,73]

^a^ISO/IEC: International Organization for Standardization/International Electrotechnical Commission.

^b^No longer covered in ISO/IEC 25010, but was part of ISO/IEC 9126-1:2001 [69], which 25010 revises.

^c^Not covered in ISO/IEC 25010.

**Table 3:** Operationalized quality aspects.

Question Number	Question	Affected quality principles
1	Has the purpose of the app been specified in the description text?	Practicality and transparency
2	Is there a description of the functions offered by the app (functionality)?	Practicality, usability, and transparency
3	Is there a description of the context and environment in which the app is to be used (application field)?	Practicality, usability, and transparency
4	Is the target group of the app (eg, doctors, students, and patients, or differently defined groups) described?	Practicality, usability, and transparency
5	Is there any indication as to whether feedback from the relevant user groups was incorporated into the design, development, or testing of the app?	Usability and transparency
6	Are there any details on where and how the app should not be used, where its limits lie (restrictions and limitations)?	Practicality, risk adequacy, and transparency
7	Are undesired effects that have already occurred been mentioned?	Risk adequacy and transparency
8	Is there a description of potential or actual risks (health, economic, and social) to which the user may be exposed when using the app?	Risk adequacy and transparency
9	Are precautions taken to avoid the above risks described?	Risk adequacy and transparency
10	Are authors or developers of the app named?	Content validity and transparency
11^a^	Is there information about the aptitude (qualification) of the authors or developers of the app?	Content validity and transparency
12	Are sources used for the app (eg, literature) named?	Content validity and transparency
13	Is it specified whether the app has been awarded certificates, quality seals or something similar by third parties?	Technical adequacy and content validity
14	Are details given with respect to quality assurance during development?	Technical adequacy and transparency
15	Is information given on whether the app is a medical device (keyword: CE label^b^)?	Legal conformity, technical adequacy, risk adequacy, and transparency
16	Is there a description of how the app is financed or who is funding it?	Content validity and transparency
17	Are conflicts of interest named (eg, involvement of an author in the app company)?	Content validity and transparency
18	Are details provided on users’ data protection rights in connection with the collection, storage, and deletion of data (eg, right to information, right of modification, right of revocation, and periods for deletion)?	Legal conformity, risk adequacy, and transparency
19	Are there any indications as to who the beneficiary(s) of the data is or are?	Legal conformity, risk adequacy, and transparency
20	Is the location where data are being stored (eg, in which country) named?	Legal conformity, risk adequacy, and transparency
21	Are there any indications of ethical innocuousness (eg, ethics vote for research apps)?	Ethical soundness, and transparency

^a^Unfortunately, question 11 was not included in the Web-based survey.

^b^Conformité Européenne. A CE labels indicates that a product sold within the European economic Area conforms to the required health, safety, and environmental protection standards.

### Evaluation Strategy

A descriptive evaluation of the frequencies, mean values, and SDs was prepared.

The primary goal of the study was to detect a change in the assessment of sufficiency for usage decisions on the basis of app description texts. The hypothesis was tested that, after confrontation with the predefined quality principles, there would be no change in the students’ assessment of the sufficiency of app descriptions for the usage decision. Bowker test of symmetry (2-sided, alpha=.05, beta=.80) [79,80] was applied and for determining effect size, Cohen g [81,82] was calculated. The aforementioned symmetry test was chosen as it provides the opportunity to test multiple nominal characteristics in associated samples. In addition, in contrast to the McNemar test, the McNemar-Bowker test is able to consider more than 2 categories.

The following points acted as secondary aims within the study:

1. Assessment of the relevance of quality principles for the usage decision.
2. Evaluation of the sufficiency of the information provided in the app descriptions to assess compliance with the quality principles.
3. Frequency of mentioned aspects as identified by the key questions in the description texts.

IBM SPSS Statistics Subscription (Build 1.0.0.1118, IBM Corporation) and R (version 3.5.1, R Core Team) [83] were used for the evaluation.

## Results

The 123 participating students regarded all 9 quality principles as “very important” or “important” (Table 4). In particular, they considered “content validity” (85.4%, 105/123) and “risk adequacy” (74%, 91/123), “legal conformity” (66.7%, 82/123) and “usability” (65%, 80/123) to be “very important.” Furthermore, the principles of “ethical soundness” (55.3%, 68/123), “practicality” (50.4%, 62/123), and “transparency” (45.5%, 56/123) were regarded as “very important” but were weaker in terms of percentage for the decision on use. The quality principles “technical adequacy” (39%, 48/123) and “resource efficiency” (34.1%, 42/123) were classified as “important” (see Table 4).

After working with the quality principles, the students were asked if they were able to determine whether these principles were met on the basis of the app descriptions (Q3, Table 5). Affirmative answers to this question were given in 31.2% (943/3087) of the evaluations of the app descriptions, with “practicality” in 71.7% (246/343) and “usability” in 39.9% (137/343) assessed as fulfilled most frequently. The worst levels of fulfillment were found for “transparency” (16.9%, 58/343) and “resource efficiency” (19.8%, 68/343). In less than one third of the app descriptions, students were able to successfully determine compliance with the quality principles “content validity” (27.1%, 93/343), “ethical soundness” (26.8%, 92/343), and “legal conformity” (22.2%, 76/343; see Table 5).

On the basis of the total number of all individual answers, participating students were unable to identify the required information in the app descriptions in 70.4% (4831/6860) of the answers (see Q4, Table 6). In 5.9% (403/6860) of the answers, students were unsure as to whether the description texts contained suitable information (“do not know”). According to the students, the greatest deficits were the lack of information on “undesirable effects” (91.8%, 315/343), “ethical soundness” (90.1%, 309/343), “risk-avoidance” (89.2%, 306/343), “conflicts of interest” (88.3%, 303/343), and “naming the data storage location” (87.8%, 301/343). Sufficient information could be found via the filter questions on the “declaration of purpose” (93.6%, 321/343) and “description of functionalities” (86.9%, 298/343). In 76.7% (263/343) of the app descriptions, assessments of the field of application could be made. However, it should be noted that only 23.3% (1600/6860) of the answers given were positive (see Q4, Table 6), corresponding only to the presence of the information necessary to answer the question in the app description.

**Table 4:** Assessment of the relevance of the 9 quality principles (Q2) for one’s own usage decision (for N=123 students).

Item	Very important, n (%)	Important, n (%)	Part/part, n (%)	Less important, n (%)	Unimportant, n (%)	Do not know, n (%)	No information, n (%)
Practicality	62 (50.4)	46 (37.4)	8 (6.5)	—^a^	2 (1.6)	5 (4.1)	—
Risk adequacy	91 (74.0)	11 (8.9)	9 (7.3)	1 (0.8)	2 (1.6)	7 (5.7)	2 (1.6)
Ethical soundness	68 (55.3)	37 (30.1)	11 (8.9)	3 (2.4)	2 (1.6)	1 (0.8)	1 (0.8)
Legal conformity	82 (66.7)	26 (21.1)	5 (4.1)	4 (3.3)	1 (0.8)	4 (3.3)	1 (0.8)
Content validity	105 (85.4)	12 (9.8)	2 (1.6)	1 (0.8)	1 (0.8)	2 (1.6)	—
Technical adequacy	3 (2.4)	48 (39.0)	47 (38.2)	3 (2.4)	—	3 (2.4)	3 (2.4)
Usability	80 (65.0)	34 (27.6)	8 (6.5)	—	—	1 (0.8)	—
Resource efficiency	36 (29.3)	42 (34.1)	24 (19.5)	10 (8.1)	3 (2.4)	8 (6.5)	—
Transparency	56 (45.5)	39 (31.7)	19 (15.4)	—	1 (0.8)	6 (4.9)	2 (1.6)

^a^No corresponding answer was given.

**Table 5:** Assessment as to whether compliance with the 9 quality principles could be determined on the basis of the available app descriptions (Q3, scale “yes,” “no,” and “do not know”), on the basis of N=343 assessments (3087 individual responses overall).

Item	Yes, n (%)	No, n (%)	Do not know, n (%)	No data, n (%)
Practicality	246 (71.7)	79 (23.0)	17 (5.0)	1 (0.3)
Risk adequacy	93 (27.1)	198 (57.7)	52 (15.2)	—^a^
Ethical soundness	92 (26.8)	211 (61.5)	40 (11.7)	—^a^
Legal conformity	76 (22.2)	231 (67.3)	36.0 (10.5)	—^a^
Content validity	93 (27.1)	210 (61.2)	37 (10.8)	3 (0.9)
Technical adequacy	100 (29.2)	199 (58.0)	41 (12.0)	3 (0.9)
Usability	137 (39.9)	179 (52.2)	25 (7.3)	2 (0.6)
Resource efficiency	68 (19.8)	205 (59.8)	69 (20.1)	1 (0.3)
Transparency	58 (16.9)	213 (62.1)	72 (21.0)	—^a^
Total number	963 (31.20)	1725 (55.88)	389 (12.60)	10 (0.32)

^a^Not applicable.

**Table 6:** Assessment of whether the 20 detailed questions could be answered on the basis of the available app descriptions (Q4, “yes”, “no”, “don't know”, based on N=343 evaluations with a total of 6860 individual answers).

Item	Yes, n (%)	No, n (%)	Do not know, n (%)	No data, n (%)
Indication of purpose	321 (93.6)	19 (5.5)	3 (0.9)	—^a^
Description of functionalities	298 (86.9)	38 (11.1)	7 (2.0)	—^a^
Information on the field of application	263 (76.7)	68 (19.8)	10 (2.9)	2 (0.6)
Information on the target group	233 (67.9)	96 (28.0)	13 (3.8)	1 (0.3)
Information on inclusion of feedback from the relevant user groups	40 (11.7)	273 (79.6)	28 (8.2)	2 (0.6)
Description of restrictions and limitations	43 (12.5)	284 (82.8)	15 (4.4)	1 (0.3)
Indication of undesired effects	8 (2.3)	315 (91.8)	18 (5.2)	2 (0.6)
Information on potential or actual risks	20 (5.8)	304 (88.6)	18 (5.2)	1 (0.3)
Information on the precautions taken to avoid the aforementioned risks	20 (5.8)	306 (89.2)	15 (4.4)	2 (0.6)
Authorship (authors or developers have been named)	67 (19.5)	249 (72.6)	25 (7.3)	2 (0.6)
Information on sources used	38 (11.1)	279 (81.3)	24 (7.0)	2 (0.6)
Information on certificates, quality seals**,** or something similar having been awarded	25 (7.3)	296 (86.3)	22 (6.4)	—^a^
Information on quality assured development	34 (9.9)	282 (82.2)	27 (7.9)	—^a^
Information on the medical device status	32 (9.3)	274 (79.9)	37 (10.8)	—^a^
Information on financing	45 (13.1)	280 (81.6)	16 (4.7)	2 (0.6)
Conflicts**-**of**-**interest**-**related information	10 (2.9)	303 (88.3)	27 (7.9)	3 (0.9)
Information about user privacy rights	41 (12.0)	277 (80.8)	23 (6.7)	2 (0.6)
Information on the beneficiary of the data	27 (7.9)	278 (81.0)	37 (10.8)	1 (0.3)
Specification of the data storage location	25 (7.3)	301 (87.8)	16 (4.7)	1 (0.3)
Information on ethical soundness	10 (2.9)	309 (90.1)	22 (6.4)	2 (0.6)
Total number of ratings	1600 (23.32)	4831 (70.42)	403 (5.87)	26 (0.38)

^a^Not applicable.

Initially, 70% (240/343) of the app descriptions were considered “sufficient” to make a decision on use. Following engagement with the quality principles, this rate was reduced to 54.2% (186/343). The proportion of app descriptions judged “insufficient” rose by an absolute value of 19.2% (66/343), from 22.7% (78/343) to 42% (144/343). The percentage of those who were undecided decreased from 7.3% (25/343) to 3.8% (13/343) and was thus almost halved (decline of 48%, 12/25; see Table 7). After the examination of quality aspects, significantly fewer assessments were considered “sufficient” than before—McNemar-Bowker Test (3)=45.803919, *P*<.001. The effect size according to Cohen was g=.295, which corresponded to a strong effect [81] (see Table 7). The calculated posthoc power was 0.99—Chi-square power calculation χ^2^_4_=0.3, *P*=.05, N=343.

Overall, 76 out of 123 students (61.8%) changed their opinion on the sufficiency of the app descriptions for a usage decision for at least 1 of the assigned apps. Of a total of 343 such assessments, 117 were revised (34.1%). A total of 90 of the 117 changes (76.9%) were corrected to a more critical assessment (changes to “insufficient” or “do not know”; see Table 8).

**Table 7:** Students’ assessment as to whether the app description text is sufficient for the usage decision. Presentation of the contingency table (Q3 vs Q5) before and after the clarification of quality principles and the targeted search for these quality criteria (yes, no, and do not know) in 343 app evaluations from 123 students.

Before information and investigation	After information and investigation			
	“Insufficient”	“Do not know”	“Sufficient”	Total number
“Insufficient”	63	2	13	78
“Don't know”	11	2	12	25
“Sufficient”	70	9	161	240
Total	144	13	186	343

**Table 8:** Presentation of the directions of change in 117 out of 343 assessments of usage decisions based on information on quality principles and criteria by 76 (61.8%) of the 123 students.

Assessments	Changes in assessment, n (%)
From “do not know” to “sufficient”	12 (10.3)
From “do not know” to “insufficient”	11 (9.4)
From “sufficient” to “do not know”	9 (7.7)
From “insufficient” to “do not know”	2 (1.7)
From “sufficient” to “insufficient”	70 (59.8)
From “insufficient” to “sufficient”	13 (11.1)
Total	117 (100.0)

## Discussion

### Principal Findings

We conducted surveys before and after confrontation with quality principles and criteria. The students evaluated the same description in both surveys. Although we did not ask to what extent the students had previous knowledge on the subject or their assigned apps (and there were no indications for this in the free text comments they were allowed to make), on the basis of our design, we were nevertheless able to determine that, after having worked with quality principles, there were indeed changes in how the participants perceived the description texts with respect to whether these possibly suffice for initial usage decisions. We were also able to obtain insights into which elements can or cannot be commonly found in the descriptions.

The study showed that, following engagement with the 9 specified quality principles (Table 2), there was a statistically significant change in the students’ assessment of the sufficiency of app descriptions for a decision on app use—McNemar-Bowker Test (3)=45.803919, *P*<.001, Cohen g=.295. In 34.1% (117/343) of the evaluations, the initial assessment was revised. Overall, more than 1 in 4 evaluations (or 3 in 4 changes of assessment) resulted in a more critical assessment. We assume that the following factors may have led to a sensitization, inciting further analytical thought when reassessing the initial question: First, the examination of app description quality by gauging the relevance of generic quality principles for the usage decision; second, the subsequent assessment of whether the description divulged the app’s fulfillment of these principles; third, the search for specific information within the texts, guided by 20 filter questions. The students rated all quality principles as “very important” or “important” for their usage decisions. In particular, “content validity” (85.4%, 105/123) and “risk adequacy” (74%, 91/123) and “legal conformity” (66.7%, 82/123) and “usability” (65%, 80/123) were “very important.” However, it was precisely these principles that the students were able to less identify with certainty in the app descriptions. It is for this reason that students were only able to assess the fulfillment of the quality criteria to a limited extent. The search for specific information in the app descriptions showed large deficits—for 16 of the 20 questions, more than 80% of the descriptions were found to contain insufficient information. In particular, statements on undesired effects (91.8%, 315/343), on the ethical harmlessness of the apps (90.1%, 309/343), on the measures taken to avoid risks (89.2%, 306/343), and regarding conflicts of interest (88.3%, 303/343) were lacking. An unspecified data storage location (87.8%, 301/343; Table 4) was also problematic.

The results allow the following conclusions to be drawn. First, when observing app descriptions, students were only able to identify a small amount of information on aspects relevant to the quality principles. This is in line with the work of other authors, in which the information content of store description texts was also evaluated as poor in terms of quality and content [84]. Second, it can be implicitly assumed that although awareness of quality principles exists, it is not generally transferred to descriptions of health apps. This is made apparent through the more critical assessment of the sufficiency of app descriptions after a sensitization to quality principles. Finally, it can be concluded that the abovementioned aspects represent essential elements for a well-founded user decision.

To form the basis for informed usage decisions, manufacturers need to provide relevant information on quality principles in an easy-to-understand manner, ideally following a universal, structured approach, easily comparable by interested parties [47]. The app description provides an ideal scope for this, as it is an obligatory requirement for all apps listed in stores on the major mobile platforms. In this study, we found that only a very small percentage of this information is made available. The specification of standardized information in the description [35] would help to solve this issue, especially if the users were to demand it. This can be achieved through the involvement of stakeholders, such as professional associations, industry associations, and consumer initiatives, that coordinate their activities across disciplines [49,50]. The message could be that manufacturers who do not include such content in the descriptions deny users the opportunity to make a well-founded decision on use. Recently, efforts have been made in various professional associations to consider compiling interdisciplinary quality criteria. Naturally, such processes are tedious because of the sheer quantity of opinions regarding the definition of the selection of criteria [50]. A process that could be concluded more quickly would be the agreement that transparency must be upheld on the part of app manufacturers and distributors.

Of course, transparency must also be appreciated and utilized by the user if a well-founded usage decision is to be made. To this end, users must become aware of their role and their individual responsibility in the (professional) use of this technology. The recognition of (professional) legal and ethical requirements of apps is not automatically conducted because of the general perception of smartphones and apps as “private matters.” In Weiser’s sense, mobile technology is already too “interwoven” with “our everyday life” [85] for it to be viewed in a differentiated way. However, the fact is that these technologies are used in professional contexts, even in health and medicine—with all their consequences. With small stakeholder campaigns and further training within the framework of the digitization debates, a great deal of sensitization could already be achieved, and a major contribution could be made in attaining the circumstances necessary for well-founded decisions on app use. Of all the solutions for evaluating apps, such as reviews, tests, certifications, and the preparation of scientific studies, app descriptions represent the first and fastest step taken by users.

### Comparison With Other Approaches in the Quality Context

There are a number of helpful and validated tools available, aiming to support those interested in health-related apps and their quality [86]. Often in the form of a checklist, these tools address various user groups and application areas, for example, Mobile Application Rating Scale [7] and user version of the Mobile Application Rating Scale [39] as well as App Chronic Disease Checklist [8]. In addition to these tools, some third-party initiatives, such as national health bodies, assign quality seals to apps or compile lists of apps they have approved. The quality of such third-party evaluations is at times questionable. How well the quality assessment processes have been designed and implemented and the scope of the assessments that are performed (eg, assessments of whether the content is adequate vs also considering technical or security-related aspects) are critical aspects when making recommendations.

In terms of this study, it was not our aim to develop yet another assessment tool for determining whether an app is of high quality. Instead, we were interested in, first, whether potentially interested parties are aware of applicable quality criteria and are able to identify corresponding information in the app descriptions, second, whether for users who have previously been unfamiliar with such criteria, a familiarization can potentially lead to changes in how they assess quality aspects, on the basis of the app descriptions. In our analysis, we found strong indications for both of these aspects. We believe that this may facilitate future evaluations on the basis of the aforementioned quality assessment tools by enabling users to more easily apply these tools.

### Limitations

#### App Selection

The inherent dependence of the quality of app selection on the quality of the search terms defined poses multiple limitations. While searching for suitable apps from the field of cardiology and pulmonology, it is possible that fitting search terms were not included or—especially with hits of partial terms—that some apps were incorrectly included. A complete (manual) screening of all apps available in the store categories “Medical” and “Health and Fitness” would not be possible because of the incredibly large volume of apps available. It is for this reason, despite limitations, that the keyword-based SARASA method [45] was used. Furthermore, it is possible that a sampling bias occurred during the selected search procedure in Apple's App Store. This is conceivable when considering the store’s category-based system, not recognizing apps falsely categorized by their manufacturers, and it may also be because of the limitation of the search to apps with German-language store descriptions, predetermined by the store front-end available for Germany. The situation may differ for App Stores available for other mobile platforms (eg, Android apps available from Google's Play Store) or even for iOS-based apps from store front-ends in other countries or apps whose store descriptions are available in other languages, which should be taken into account in subsequent investigations.

In addition, the SARASA method led to a variable selection of apps that were probably not directly comparable because of their different application areas and target groups. Nevertheless, we believe that this variability was more a strength than a weakness of our evaluation, as we were not interested in the direct comparability of apps but rather in the evaluation of quality aspects in a typical setting. This is given as users are able to obtain apps using keyword-based searches in the store.

#### Study Population

It may also be argued that our participants’ demographics are not fully representative of the German population, for example, with respect to their age, level of education, and smart device usage patterns, with almost 59% (72/123; Table 1) of the participants stating that they were using iOS-based devices versus only about 23% market share for such devices in the German population in December 2018 [87]. Despite these discrepancies, the study population reflects the often-mentioned greater popularity of the iOS platform among those working in the medical field [88], and thus the participants may well prove to be a representative sample, at least in comparison to their future colleagues. Platform-related effects on our results were probably negligible, as the students were requested to solely consider the provided store description texts, without platform specifics, and not the apps themselves. Moreover, it has been shown that there are only small differences among users of various mobile platforms, if sociodemographics are accounted for [89].

Another possible limitation regarding our choice of students as the study population may be the students’ lack of experience in the medical field and their lack of exposure to the quality aspects investigated, potentially making it more difficult for them to assess the content of the app descriptions. Upon reflection, we believe this had little, if any, influence. As app descriptions are commonly written not to convey detailed, in-depth information, but rather to satisfy marketing requirements—after all, manufacturers hardly have a chance to restrict who has access to them—one would expect that only in rare circumstances would the information conveyed in descriptions require knowledge surpassing that of fourth year medical students. In addition, in a previous study [45], for a somewhat similar selection of apps, we applied automated algorithms for text complexity to the descriptions, with calculations based on sentence length, number of syllables, etc, to determine the level of education necessary for reading comprehension. In that case, for about 3 quarters of the apps, a level of high school education or less would have been sufficient for comprehension. We therefore believe that medical students, who are as far along in their studies as our fourth year participants, should have sufficient medical background and reading proficiency to perform basic checks of medically-oriented app descriptions. Moreover, an objection that students do not have the knowledge necessary for basic assessments of usability and information security can hardly be raised. For today’s students, a majority of which have grown up with information technology and could therefore be considered “digital natives,” at least a basic understanding of these aspects can be assumed. In any case, to be truly meaningful, expert-level assessments would require in-depth analyses of the apps themselves rather than an evaluation of store description texts.

#### Questionnaire Design

The questionnaires were pretested with 4 medical students from different semesters. It would have been sounder to test with a population comparable with the target group. Unfortunately, appropriate candidates could not have been recruited without provoking a bias (prospective course participants), which is why we refrained from doing so. The pretest was conducted without any evidence of comprehension problems when paraphrasing so that the authors saw no reason for any changes. Despite this, some of the questionnaires within the study were not fully completed. A dropout analysis was not carried out for reasons of capacity; however, it is planned for subsequent rounds.

Unfortunately, the filter question “Is there information about the aptitude (qualification) of the authors/developers of the app?” was not included in the Web-based survey, although this was planned. This will be done in a subsequent study, as determining whether the authors’ and developers’ qualifications befit the purpose of the app may be of interest—appropriate qualifications can be a surrogate parameter for the quality of the content. If those involved are experts in the respective field, be it because they obtained an academic degree or another type of suitable qualification, it is more likely that the content will be valid and of high quality than if it was written by others who are not similarly educated.

It would also have been desirable to discriminate between apps in general and health-related apps when asking participants to assess the importance of quality criteria. In addition to the general review of the quality principles, this would have made it possible to assess whether the participants’ perceptions of quality criteria differ between general and particularly sensitive health contexts.

### Outlook

Planned follow-up studies should aim to confirm and extend the results of this study. A more diverse study population (larger number of participants, other academic years, other health-related programs, and vocational training) should be included. On the whole, it is most important to facilitate analyses that can quantify the relevance of the individual quality principles and their contribution to the assessment process. This can be achieved by creating a larger database through experiment reproduction. Through this process, the isolation of a truly necessary and sufficient number of principles would be better possible. The operationalization of the quality principles will be examined in a separate paper. The aim is to identify potential candidates from the existing set of known criteria, to check their suitability and, if necessary, to synthesize new criteria. A time series, for example, through yearly evaluations in similar classes, possibly at other universities, could also be potentially used to determine whether, and if so, how, students’ awareness and perception of quality criteria in health-related app contexts change over time.

### Conclusions

To provide users with orientation and to strengthen their decision-making competence, the app description texts must contain significantly more relevant information, for example, by including information compiled by following a standardized and comprehensive structure [19,35]. App stores should encourage this approach, as it would significantly aid in satisfying their users’ need for information. However, whether (possibly mandatory) validations or cross checks of the provided information by independent experts, for example, before publication of a health-related app in an app store, would encourage trust and actually benefit users or would rather impede innovations seems questionable. Serious checks performed by experts in the respective field would—because of the steadily growing number of apps—require a significant number of experts to be able to perform these checks in a timely manner and would also introduce costs that many (at least smaller or startup) manufacturers would be unable or unwilling to bear. We therefore believe that sensitizing users to the importance of applying quality principles to any information available about an app, including app descriptions, will be much more effective.

## Abbreviations

CE: Conformité Européenne
ISO/IEC: International Organization for Standardization/International Electrotechnical Commission
SARASA: Semiautomated Retrospective App Store Analysis
